# Tuberosity reconstruction baseplate for shoulder hemiarthroplasty: Morphological design and biomaterial application

**DOI:** 10.3389/fbioe.2022.1047187

**Published:** 2022-10-28

**Authors:** Zhentao Ding, Jiabao Ju, Mingtai Ma, Yichong Zhang, Jianhai Chen

**Affiliations:** ^1^ Department of Orthopedics and Trauma, Peking University People’s Hospital, Beijing, China; ^2^ National Center for Trauma Medicine, Peking University People’s Hospital, Beijing, China; ^3^ Key Laboratory of Trauma and Neural Regeneration (Peking University), Ministry of Education, Beijing, China

**Keywords:** tuberosity reconstruction baseplate, shoulder hemiarthroplasty, tuberosity healing, carbon fiber reinforced polymer, finite element analysis, structure optimization

## Abstract

**Background:** Shoulder hemiarthroplasty is prone to tuberosity malposition and migration, reducing the rate of tuberosity healing. We proposed to design a tuberosity reconstruction baseplate to assist in tuberosity integration and to evaluate the mechanical properties of baseplate made from the novel biomaterial carbon fiber reinforced polymer (CFRP) composites.

**Methods:** The three-dimensional model of native proximal humerus was constructed by computed tomography (CT) data. The morphological design of baseplate was based on the tuberosity contour and rotator cuff footprint. Finite element models were created for different thicknesses of CFRP composites, poly (ether-ether-ketone) (PEEK) and titanium-nickel (TiNi) alloy. The permissible load and suture hole displacements were applied to evaluate the mechanical properties.

**Results:** The structurally optimized model made of CFRP composites provided superior strength and deformability, compared to the PEEK material and TiNi alloy. Its permissible load was above 200 N and the suture hole displacement was between 0.9 and 1.4 mm.

**Conclusion:** This study proposed a method for designing tuberosity reconstruction baseplate based on morphological data and extended the application of biomaterial CFRP composites in orthopedics field. The optimized model made of CFRP composites allowed a certain extent of elastic deformation and showed the possibility for dynamic compression of tuberosity bone blocks.

## Introduction

Proximal humeral fractures are the third most common type of osteoporotic fracture in elderly patients (Launonen et al., 2015). Older patients often present with comminuted Neer 3- and 4-part fractures and are vulnerable to complications after plate fixation (Stone and Namdari, 2019). Nowadays, shoulder hemiarthroplasty is an effective treatment option for non-reconstructable proximal humeral fractures. However, recent clinical follow-up studies have demonstrated that the recovery of mobility and function after hemiarthroplasty is not satisfactory (Yahuaca et al., 2020; Amundsen et al., 2021). Nonunion and malunion of tuberosities are risk factors for postoperative joint function (Tanner and Cofield, 1983; Kralinger et al., 2004). Therefore, the focus of hemiarthroplasty is on restoring anatomical structure of the proximal humerus.

Tuberosity malposition is associated substantially with the implant design and fixation technique. After implantation of the humeral prosthetic stem, the tuberosity fracture fragments are repositioned by suture traction, and then the greater and lesser tuberosity are reattached to the stem by cerclage (Neer, 1970). However, the cerclage suture is prone to inferior migration of tuberosities, which leads to malreduction (Boileau et al., 2002). Furthermore, due to the secondary migration following rotator cuff contraction, the cerclage suture is difficult to achieve effective tuberosity reconstruction (Grubhofer et al., 2021). In the setting of bony comminution and local osteoporosis, there is also lack of anatomical landmark for tuberosity reduction (Frankle et al., 2001).

In the light of these issues, we propose to design a tuberosity reconstruction baseplate placed between the prosthetic humeral head and stem to assist in tuberosity reduction and fixation. The contour of greater and lesser tuberosity designed on this baseplate serves as a landmark for anatomical reduction. Prefabricated suture holes corresponding to the rotator cuff insertions are available to determine relative position of the prosthetic humeral head and tuberosities. In addition, the baseplate is expected to have a certain elastic deformability. With continuous traction of the rotator cuff, the baseplate is able to provide dynamic compression between the tuberosity bone blocks by tension band effect. In this study, the materials for baseplate were screened by finite element analysis, including the novel biomaterial carbon fiber reinforced polymer (CFRP) composites [CF/epoxy laminates, Toray Company Ltd., material code T800/3900 (Ahmad et al., 2019)] and two other conventional orthopedic implant materials. Subsequently, the strength and deformability of baseplate were improved through structural optimization.

## Materials and methods

### Tuberosity reconstruction baseplate design

The geometry of tuberosity reconstruction baseplate consists of the greater and lesser tuberosity and intertubercular groove that provides a landmark for the anatomical reduction in hemiarthroplasty procedure. The morphological design requires a suitable section to depict the humeral shape of anatomical neck and its distal region. The anatomical neck section is approximately circular and does not allow for a distinct tuberosity contour. Therefore, the baseplate design should be based on the distal section of anatomical neck.

**FIGURE 1:**
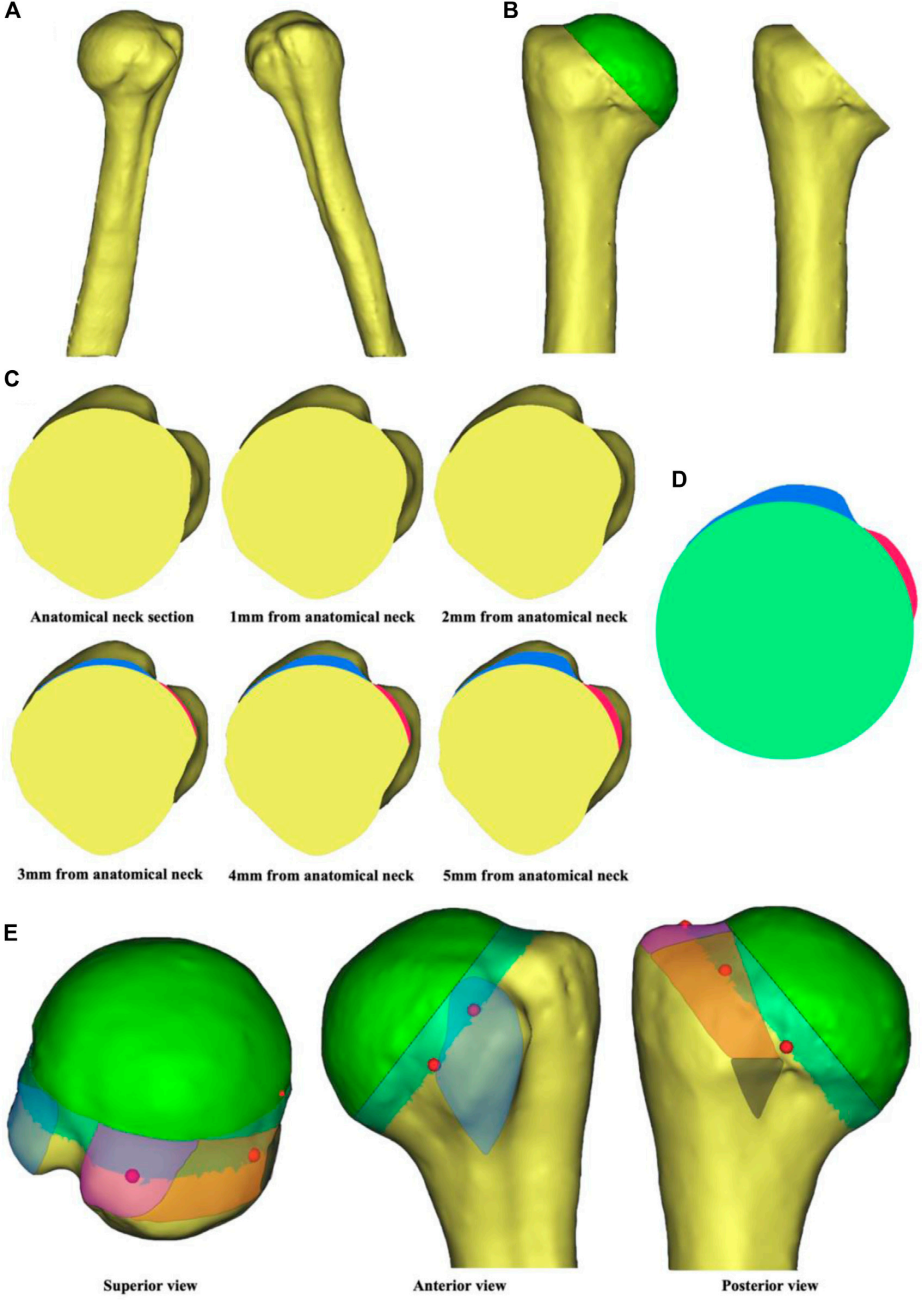
Steps in tuberosity reconstruction baseplate design. **(A)** Construction of a 3D native proximal humerus model. **(B)** Virtual osteotomy along the anatomical neck. **(C)** Anatomical neck section and sections 1–5 mm from anatomical neck. The blue region represents contour of the greater tuberosity and the red region represents contour of the lesser tuberosity. **(D)** Morphological design of the baseplate. **(E)** Location of the suture holes based on rotator cuff footprint. The blue region is for subscapularis, the purple region is for supraspinatus, the orange region is for infraspinatus, and the black region is for teres minor. The red dots are reference points for the location of suture holes.

A 47-year-old male volunteer (Ethics Committee of Peking University People’s Hospital, 2020PHB072-01, Beijing, China) with no severe trauma history or obvious anatomical abnormality was recruited. Computed tomography (CT) data of the proximal humerus were imported into Mimics 19.0 (Materialise, Leuven, Belgium) in DICOM format. Automatic threshold-based segmentation extracted bone tissue from the CT data to construct a 3D model of the native proximal humerus (Figure 1A). In this model, the contour of anatomical neck could be clearly identified. The osteotomy level was set at a 45-degree angle to the humeral stem, and the humeral head was virtually resected along the anatomical neck to obtain the anatomical neck section (Figure 1B). The virtual osteotomy level was translated distally to obtain sections 1–5 mm from anatomical neck (Figure 1C). Among these, section 5 mm from anatomical neck showed a clear contour of the greater and lesser tuberosity as well as intertubercular groove. Therefore, this section was chosen for the morphological design (Figure 1D). According to the anatomical landmarks of proximal humerus, the rotator cuff footprint was depicted on the 3D model (Figure 1E) (Curtis et al., 2006; Mochizuki et al., 2008). Prefabricated suture holes were determined at the corresponding locations on baseplate. The suture holes were set at 2 mm in diameter and 2 mm from the edge. Each suture hole was spaced more than 1 cm apart.

**FIGURE 2:**
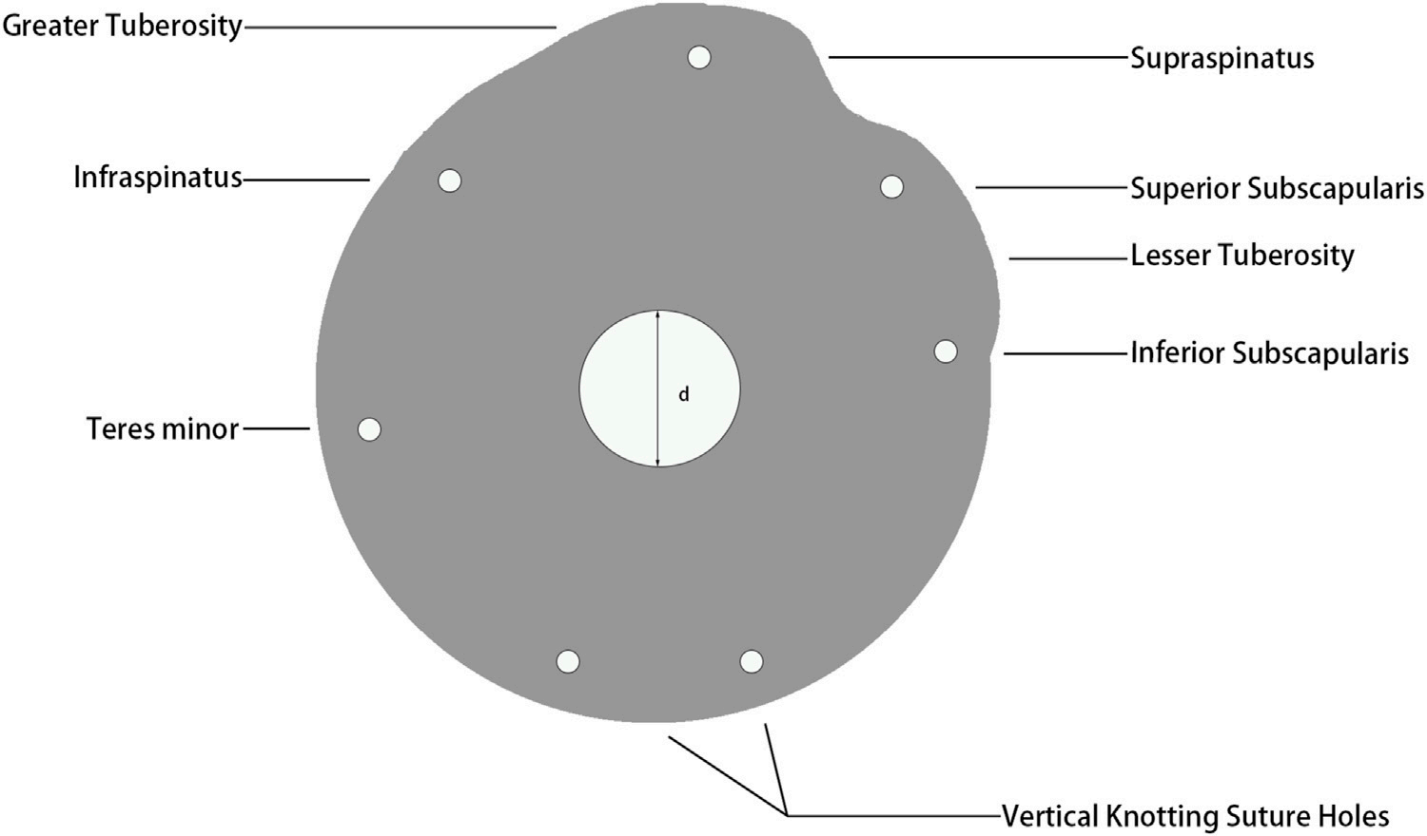
Blueprint of tuberosity reconstruction baseplate.

The blueprint of tuberosity reconstruction baseplate was shown in Figure 2. Two prominences corresponded to the greater and lesser tuberosity. Five suture holes adjacent to the tuberosities corresponded to superior and inferior subscapularis, supraspinatus, infraspinatus, and teres minor, respectively, for knotting and fixing the baseplate to rotator cuff. Two additional holes were provided at 6 o’clock position for vertical knotting. The baseplate thicknesses were set to 1, 1.5, 2, 2.5, and 3 mm, and the diameter was set to 40 mm to create five finite element models.

### Finite element analysis

The finite element models were automatically meshed in Hypermesh 12.0 (Altair Engineering GmbH, Böblingen, Germany). The mesh type was a 4-node linear tetrahedral element (C3D4) with the mesh size of 0.4 mm. Preprocessing and linear static analysis were performed in Ansys 19.0 (ANSYS, Inc., Canonsburg, PA, United States). The baseplates were manufactured from three of the most widely used orthopedic implant materials, including poly (ether-ether-ketone) (PEEK), titanium-nickel (TiNi) alloy and the novel biomaterial CFRP composites. The PEEK and TiNi alloy were set to be homogeneous and isotropic. The CFRP composites consisted of multiple layers stacked in different directions with quasi-isotropic properties. Each of the layers was 0.1875 mm thick and the stacking sequence was [45/0/−45/90]_s_. The Young’s modulus, Poisson’s ratio, density and yield strength of three baseplate materials were presented in Table 1 (Song et al., 2000; Ahmad et al., 2018; Vogel et al., 2018). In addition, the shear modulus of CFRP composites was set at 3,000 MPa. None of these baseplates exceeded the yield strength and were therefore modelled as linear elastic.

**TABLE 1:** Mechanical properties of baseplate materials.

Material	Young’s modulus (MPa)	Poisson’s ratio	Density (kg/m^3^)	Yield strength (MPa)
PEEK	3,450	0.40	1,300	95
TiNi	83,000	0.33	6,450	443
CFRP	E_1_ = 100,000	µ_1_ = 0.40	1,550	1,000
	E_2_ = 5,000	µ_2_ = 0.30		

Due to the complex actual loading conditions of rotator cuff, loads of the same magnitude were applied vertically at seven suture holes to simulate extreme situations. The central hole was constrained. Firstly, a 1N load was applied at each suture hole to obtain the maximum von Mises stress by finite element analysis. And then the permissible load was calculated from the yield strength of materials. With the permissible load applied, the displacements of five suture holes corresponding to rotator cuff in each finite element model were compared.

*In vivo*, the maximum force of a single rotator cuff muscle does not exceed 200 N (Meyer et al., 2018). Also, many of the high-strength sutures available for rotator cuff repair have a failure strength above 200 N (Borbas et al., 2021). Therefore, when the permissible load exceeds 200 N, the baseplate only produces elastic deformation in practice and does not yield. In addition, if a certain extent of displacement occurs in the suture holes corresponding to rotator cuff, it is possible for the baseplate to perform as a tension band to achieve dynamic compression between tuberosities.

### Structure optimization

Based on the finite element analysis of three baseplate materials, the model with higher permissible load and larger suture hole displacement could be selected for further structure optimization. During the optimization process, thicker materials or more layers were designed at the von Mises stress concentrations to increase the permissible load, while thinner materials or fewer layers were designed in other regions to enhance the deformability. A stress of 200N was loaded vertically on each suture hole of the hybrid design model. The maximum von Mises stress and suture hole displacements were recorded.

## Results

### Stress distribution and displacement

**FIGURE 3:**
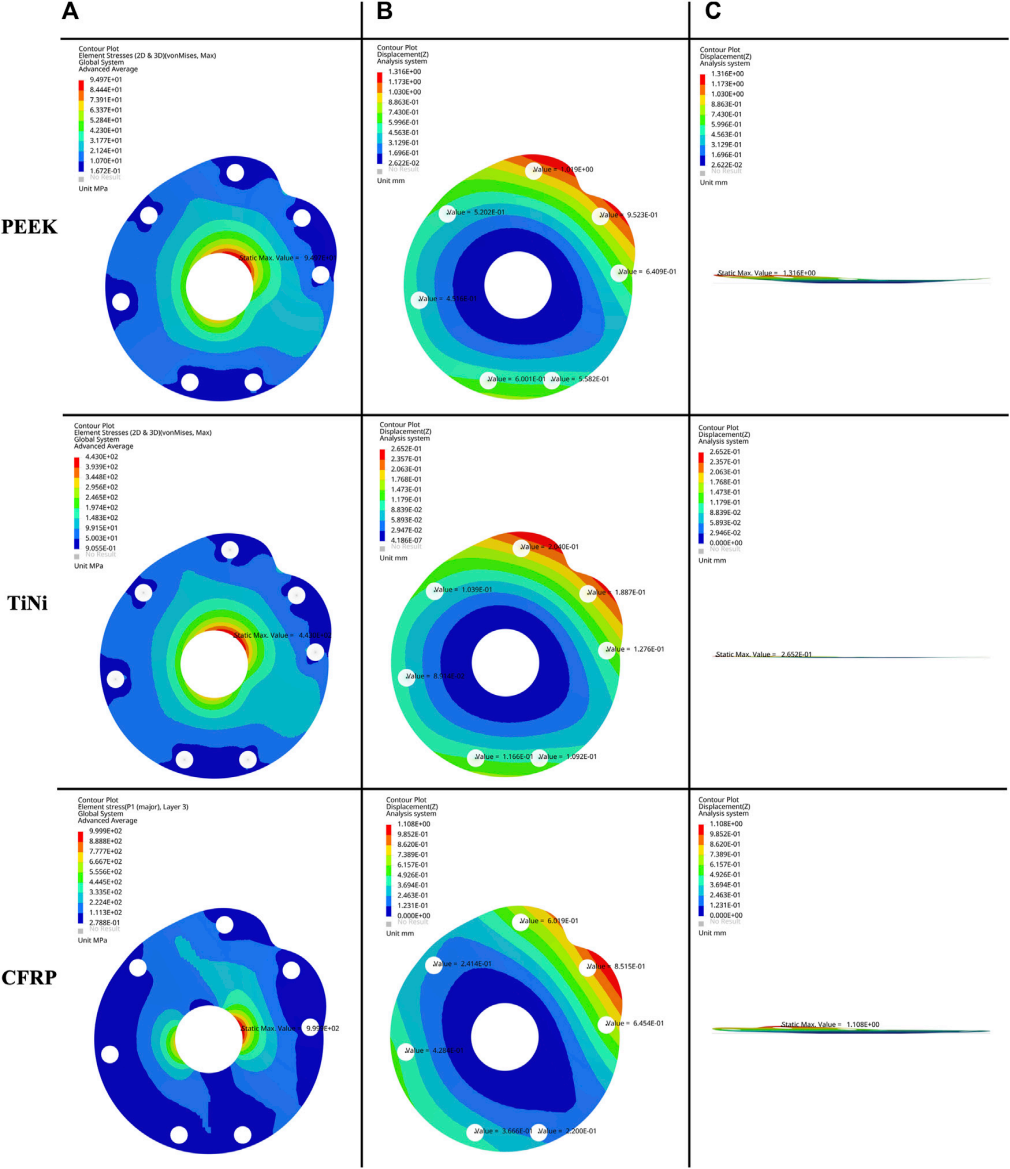
**(A)** Frontal view of von Mises stress nephogram, **(B)** frontal view and **(C)** lateral view of displacement nephogram for a 2 mm thick baseplate model under permissible load.

The von Mises stress nephogram (Figure 3A) and displacement nephogram (Figure 3B,C) for three baseplate materials with a thickness of 2 mm showed similar distribution pattern. The von Mises stress was concentrated around the central hole, with the highest stress from 12 o’ to 3 o’clock position, followed by 6 o’–9 o’clock. The displacement was largest at the suture holes corresponding to supraspinatus and superior subscapularis.

The permissible loads and suture hole displacements for three baseplate materials with five thicknesses were demonstrated in Table 2. As the baseplate thickened, the permissible load increased and the suture hole displacement decreased. For the same thickness, the baseplate made of PEEK had a lower permissible load and a higher suture hole displacement. The baseplate made of TiNi alloy had a higher permissible load and a lower suture hole displacement. The baseplate made of CFRP composites had the highest permissible load and a similar displacement to the PEEK material.

**TABLE 2:** Permissible load and suture hole displacement of finite element models.

Material	Thickness (mm)	Permissible load (MPa)	Suture hole displacement (mm)
PEEK	1.0	9.3	0.8–1.9
	1.5	21.0	0.6–1.3
	2.0	37.4	0.5–1.0
	2.5	58.8	0.3–0.8
	3.0	85.0	0.3–0.7
TiNi	1.0	43.0	0.2–0.4
	1.5	96.9	0.1–0.3
	2.0	172.7	0.1–0.2
	2.5	270.8	0.1
	3.0	391.3	0.1
CFRP	1.0	67.8	0.8–1.4
	1.5	103.2	0.7–1.4
	2.0	211.4	0.4–0.9
	2.5	396.7	0.4–0.8
	3.0	407.3	0.2–0.8

### Structure optimization

**FIGURE 4:**
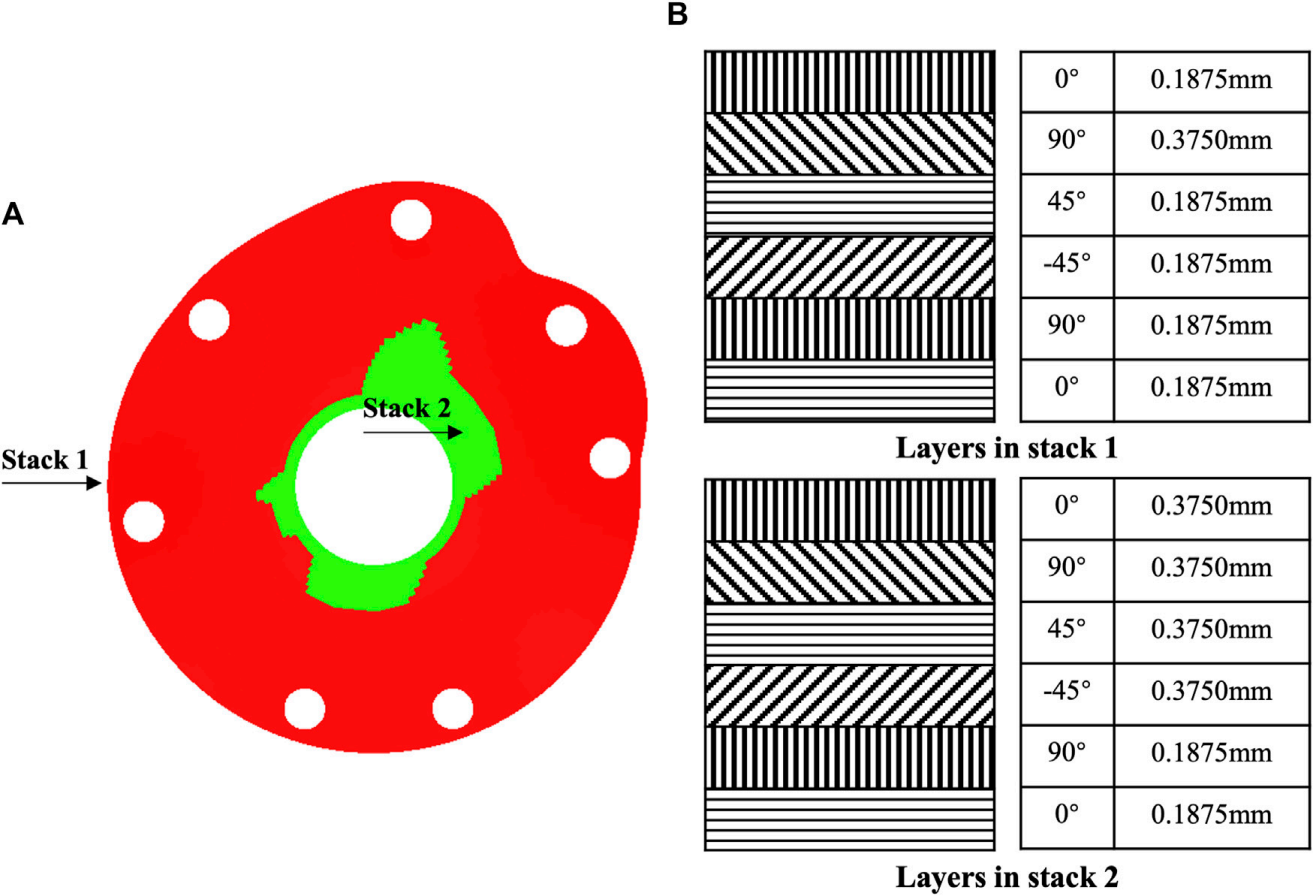
Structure optimization of tuberosity reconstruction baseplate. **(A)** Hybrid structural design and **(B)** stack design.

The CFRP baseplates with thicknesses of 1.5 and 2 mm provided displacements up to 0.7–1.4 and 0.4–0.9 mm respectively, which could serve as base models for structure optimization (Table 2). In the optimized model with hybrid design, two different stacking methods with different thicknesses were designed (Figure 4A), each with six layers (Figure 4B). Stack 1 was located in the peripheral region with a thickness of 1.3125 mm. Stack two was located around the central hole, mainly from 12 o’clock to 3 o’clock and from 6 o’clock to 9 o’clock. Stack 2 had a thickness of 1.875 mm.

**FIGURE 5:**
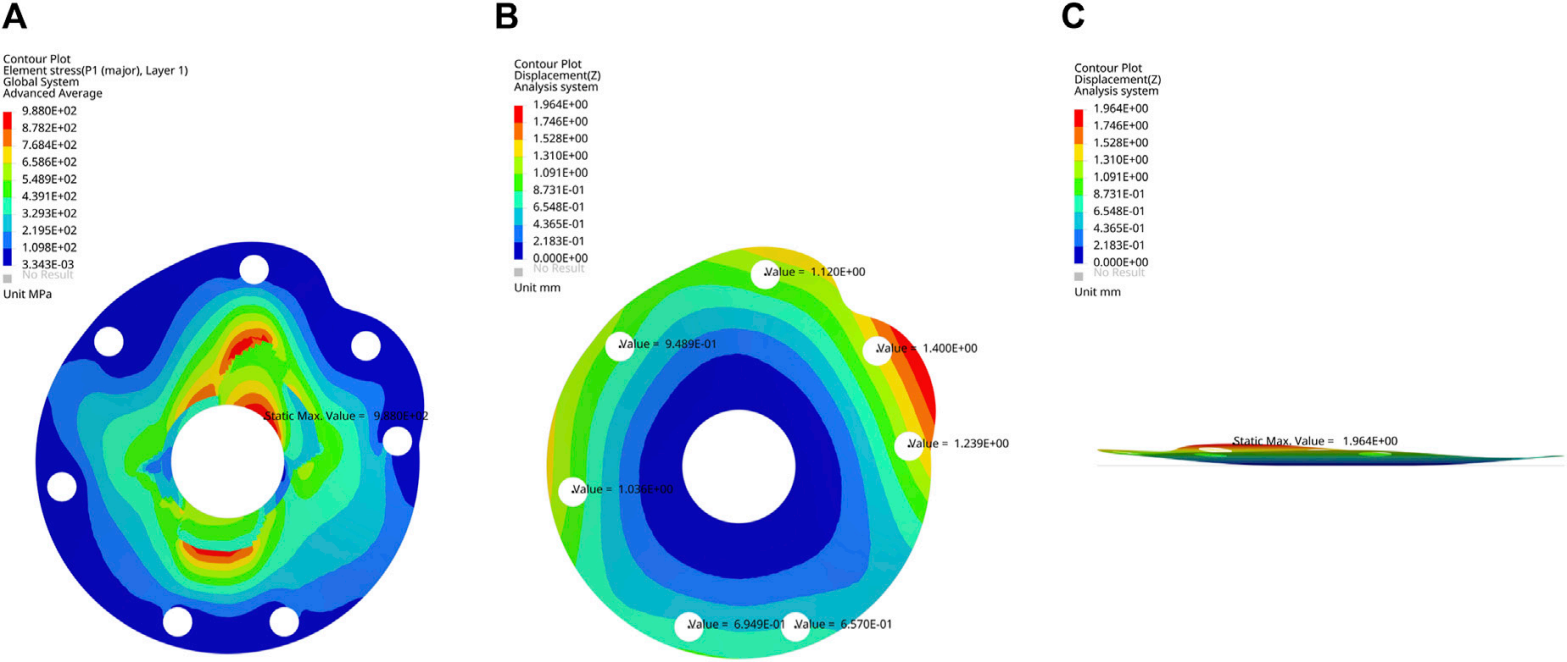
**(A)** Frontal view of von Mises stress nephogram, **(B)** frontal view and **(C)** lateral view of displacement nephogram for the optimized baseplate model under 200 N load.

The maximum von Mises stress of the structurally optimized model was 988 MPa when 200 N load was applied at each suture hole, which was lower than the yield strength of CFRP composite material (Figure 5A). At the greater tuberosity, the suture hole displacements were 1.1, 0.9 and 1.0 mm for the supraspinatus, infraspinatus and teres minor, respectively (Figure 5B). At the lesser tuberosity, the suture hole displacements were 1.4 and 1.2 mm for the superior and inferior subscapularis, respectively (Figure 5B). In comparison, the optimized model presented higher displacements for suture holes. The overall deformation of baseplate was visualized through the lateral view of displacement nephogram (Figure 5C).

## Discussion

This study presented a feasible method to design a tuberosity reconstruction baseplate based on CT data of the native proximal humerus for application in shoulder hemiarthroplasty. Three commonly used orthopedic implant materials and five thicknesses were screened through finite element analysis. Among these, an optimized model with hybrid design made of CFRP composites demonstrated higher permissible load and larger suture hole displacements, with the potential to achieve anatomical reduction and dynamic compression of tuberosities.

The function of shoulder joint after hemiarthroplasty is highly dependent on the tuberosity healing and therefore the tuberosity reconstruction has received a lot of attention (Simovitch et al., 2019). Doursounian et al. (2019) passed two high-strength sutures through the subscapularis and infraspinatus insertions and knotted them to achieve horizontal fixation of tuberosities, then passed two more sutures sequentially through drilled holes on the humeral shaft and the supraspinatus insertion to achieve vertical fixation with a figure-of-eight tension band. Onggo et al. (2021) found that fracture stems significantly improved the healing rate of greater tuberosity compared to nonfracture stems, possibly due to the lateral fin providing a platform for tuberosity reconstruction. Krause et al. (2007) used steel cable cerclage to enhance fixation stability and significantly reduced the incidence of tuberosity migration and resorption. However, these methods did not overcome the inherent shortcomings of cerclage. Cerclage suture tends to result in low tuberosity reduction and there is no continuous compressive stress between the fracture fragments (Cadet and Ahmad, 2012). As the edema resolves, the fracture fragments separate under the traction of rotator cuff, eventually appearing as scar formation rather than bony healing (Grubhofer et al., 2021).

Due to the unpredictability of tuberosity healing in hemiarthroplasty, more surgeons prefer to reverse total shoulder arthroplasty (RTSA) for complex proximal humeral fractures (Panagopoulos et al., 2022). However, the revision of RTSA is still a challenge, so the hemiarthroplasty remains an irreplaceable treatment option at present (Schultz et al., 2021). The main features of tuberosity reconstruction baseplate include the provision of landmarks and suture holes for anatomical reduction and a potential tension band effect based on elastic deformability. Therefore, the baseplate offers a promising alternative technique to tuberosity suture augmentation.

The results of this study showed that the PEEK material had lower strength but superior deformability. The TiNi alloy had higher strength but inferior deformability. The CFRP composite material, in contrast, provided the highest strength with similar deformability to the PEEK material. The CFRP composites are commonly applied in the automotive and aerospace industries as a high strength-to-weight ratio material. In recent years, CFRP composites has been applied increasingly in orthopedics field, gradually replacing traditional metal-based implants (Merolli, 2019; Lin et al., 2020). In the CF/epoxy composites chosen for baseplate, the carbon fibers with moderate elastic modulus and high tensile strength are reinforced with epoxy resin (Ahmad et al., 2019). The presence of epoxy resin adversely affects the shear modulus, but the increased deformability meets the mechanical requirements of baseplate (Mahboob et al., 2021). Moreover, hybrid design is a popular technique for structural optimization (Ghosh et al., 2022). This technique maximizes the deformability of baseplate while maintaining its mechanical strength.

*In vitro* biomechanical experiments found that the maximum contraction force of supraspinatus muscle was approximately 302 N (Burkhart, 2000). Meyer et al. (2018) performed high voltage electrical stimulation on the suprascapular nerve during rotator cuff repair surgery and discovered that the maximum contraction force of single rotator cuff muscle did not exceed 200 N. The loading conditions of rotator cuff in daily activities is complicated and many patients are unable to achieve the theoretical maximum contraction. Furthermore, the strength of rotator cuff muscles varies in different positions (Tsuruike et al., 2021). Thus, in this study we only used simplified loading configurations to screen baseplate materials and thicknesses by simulating extreme conditions. This may lead to an overestimation of baseplate deformability.

There are other limitations to this study. There are variations in tuberosity geometry in different populations, but only data from a single volunteer were utilized as the design basis. In addition, there is no definite evidence to suggest whether a displacement of 0.9–1.4 mm in the optimized model enables the dynamic compression effect. The next step is to validate the mechanical properties of baseplate in cadaveric and mechanical experiments.

## Conclusion

This study proposed the concept of tuberosity reconstruction baseplate and provided a design procedure based on CT data of the native proximal humerus. A structurally optimized model made of CFRP composites presented the potential to realize anatomical reduction and dynamic compression of tuberosities. As a novel biomaterial, the mechanical properties and clinical efficacy of CFRP composites need further evaluation.

## Data Availability

The raw data supporting the conclusion of this article will be made available by the authors, without undue reservation.
